# Health risk management framework for heavy metals and cyanide in Kwekwe city of Zimbabwe: a mixed-method study protocol

**DOI:** 10.1186/s41043-023-00367-5

**Published:** 2023-04-03

**Authors:** Sheunesu Ngwenya, Ntsieni S. Mashau, Emmanuel S. Mhlongo, Afsatou N. Traoré, Azwinndini G. Mudau

**Affiliations:** 1 Department of Public Health, School of Health Sciences, University of Venda, P Bag X5050, Thohoyandou, South Africa; 2 Department of Earth Sciences, University of Venda, Thohoyandou, South Africa; 3 Department of Biochemistry and Microbiology, University of Venda, Thohoyandou, South Africa

**Keywords:** Cancer risk, Delphi technique, Health risk, Kwekwe, Mixed-method study, Potentially toxic metal, Purposive sampling

## Abstract

**Background:**

According to WHO, in 2015, over 35% of ischaemic heart disease, the leading cause of death and disability worldwide, and about 42% of strokes, the second largest contributor to global mortality, could have been prevented by reducing or removing exposure to chemical pollutants. Heavy metal and cyanide pollution are prevalent in developing countries, especially in sub-Saharan Africa where the effects of industrial pollutants are more severe, partly due to poor regulation. In Zimbabwe, the mining industry alone contributed to 25% of occupational conditions and injuries in 2020. Therefore, to mitigate these problems, this study seeks to develop a health risk management framework for heavy metals and cyanide pollution in the industrial city of Kwekwe.

**Methods:**

The convergent parallel mixed-method study design will be utilised. Qualitative and quantitative data will be collected, analysed, and merged in order to inform the development of the risk framework. An analytical cross-sectional survey would be used to determine levels of heavy metals in surface water, soil, and vegetables. Free cyanide will be determined in surface water samples only. The phenomenological qualitative inquiry will be used to investigate health events and risks associated with potentially toxic pollutants (heavy metals and cyanide) to describe or interpret participants' lived experiences. The qualitative and quantitative results will be used to develop and validate the framework to manage identified health risks. For data analysis, statistical analysis will be used in the quantitative study, while thematic analysis will be used in the qualitative study. The study was approved by the University of Venda Ethics Committee (Registration Number FHS/22/PH/05/2306) and the Medical Research Council of Zimbabwe (Approval Number MRCZ/A/2944). All ethical principles will be adhered to throughout the study in accordance with the Helsinki Declaration.

**Discussion:**

While existing risk management frameworks have significantly contributed to human and environmental health protection, novel and comprehensive frameworks need to be developed to counter the ever-dynamic and evolving risks associated with chemical pollutants. If the management framework is successfully developed, it could offer an opportunity for the prevention and control of potentially toxic elements.

## Abstract (summary)

In Zimbabwe, just like most developing countries, the problem of environmental pollution continues to threaten and endanger public health. Current pollution management measures seem insufficient in combating the problem to the extent that communities living in or near industrial areas continue to suffer from associated acute and chronic health conditions. Kwekwe city is an industrial and mining area with several abandoned and active mines and processing plants. Artisanal mining is widespread in the area, and many parts of the city's landscape are littered with open mining pits, dumps, and abandoned tailings. Most of these facilities are potential sources of heavy metals and cyanide and pose risks to human health. This study, therefore, seeks to develop a health risk management framework to address problems, which are associated with anthropogenic pollutants such as heavy metals and cyanide. The study will be conducted in two phases, that is, the first phase would involve the collection and analysis of empirical data from environmental samples, residents, and key stakeholders on heavy metal and cyanide pollution and associated health risks. The second phase would utilise findings from the first phase to develop a regulatory framework and to manage identified health risks. The developed framework would be validated through stakeholder and expert participation. The developed health risk management framework could be useful in the prevention and control environmental contamination, hence assisting to improve non-communicable diseases’ outcomes in Kwekwe city.

## Background

Approximately nine million people died in 2015 due to environmental pollution, a toll greater than the total deaths attributed to HIV and AIDS, malaria, and tuberculosis [1]. Over 35% of ischaemic heart disease, the global leading cause of death and disability, and 42% of strokes, the second largest contributor to global mortality, could have been prevented by reducing or avoiding exposure to chemical pollutants [2]. Several studies have shown that heavy metal and cyanide contamination of the environment pose serious health risks [3, 4]. Heavy metals in urban sediments, water, food, air, and other media pose both non-carcinogenic and carcinogenic risks [5–9]. Elevated levels of heavy metals have been shown to a strong correlation with stunted growth among local people living in or near mining areas [10]. Most of the health risk management frameworks have been developed at national level in developed countries (USA, Australia, and Canada) and are general frameworks for managing chemical, biological and physical risks [4, 11]. The majority of studies on heavy metals made recommendations to prevent and control environmental pollution but did not develop health risk management frameworks. The problem of heavy metals and cyanide pollution is more pronounced in developing countries, especially in sub-Saharan Africa where the effects of industrial pollutants are more severe [12, 13]. Several studies in the region found a correlation between living near industries and health conditions associated with nickel (Ni), chromium (Cr), arsenic (As), zinc (Zn), copper (Cu), lead (Pb), mercury (Hg), and cadmium (Cd) exposure [12, 14]. People living near mines and industries suffered from diseases such as asthma, cough and emphysema [12, 14]. Artisanal gold miners exposed to mercury poisoning experienced neurological disorders and birth defects [15]. Cyanide (CN) contamination and intoxication problems are common in poor and third-world countries where artisanal gold mining is prevalent. Elevated levels of CN were found in some agricultural produce, and this posed adverse non-carcinogenic risks to consumers [16, 17].

In Zimbabwe, the artisanal gold mining sector employed 1.5 million workers, while the whole mining sector contributed 16% to the national gross domestic product from mineral exports, which accounted for 60% of total export earnings in 2020 [18]. However, 25% of the occupational conditions and injuries in 2020 emanated from the mining industry. Peer-reviewed studies on environmental pollution in Zimbabwe did not develop regulatory frameworks or schemes to prevent and control health risks associated with pollutants. Most studies focused on the determination of levels of environmental contaminants, pollutant spatial distribution, pollution indices, statistical analysis, and health risk assessments. Zimbabwe as country does not have a national health risk management framework and is ranked among the world's top ten countries that use large quantities of chemicals to extract gold [15]. Mercury levels were found to be high in artisanal miners' blood, urine, and hair [15]. Also, high levels of aluminium (Al), Zn, and Pb were found in mushrooms and their substrates in a mining town and forests [19], while the spatial distribution of Cu, Cr, Pb, and Cr was significantly high in sediments. A study on water pollution found levels of Pb to be above WHO and Standards Association of Zimbabwe (SAZ) limits, and the metal had the potential to pose health risks for people living near or along a river [20]. Levels of Cd, Pb, and Hg in water were found in other studies in Zimbabwe [21, 22]. Although levels of these heavy metals were found to be above Environmental Management Agency (EMA), WHO, and SAZ standards, no health risk assessment was carried out in these studies. Authors just made management, legal, and policy recommendations to manage the problem. This study seeks to develop a management framework to prevent and control potentially toxic metal and cyanide health risks in order to protect and promote public health. The target contaminants, which are associated with industrial and mining activities in the study area, include Cd, Cr, Cu, Hg, Pb, Fe, Zn, and CN. In this study, a health risk assessment would be a critical tool for estimating health effects or risks that result from exposure to carcinogenic and non-carcinogenic chemicals [12, 23, 24].

Several methods used for health risk assessment include the US EPA method; CombiTool, a computer program for the analysis of toxic effects of environmental mixtures; DALYs method; and the EuroMix method for mixture risk assessment among others [24–26]. In this study, the USEPA method will be used for risk assessment and has four steps. Hazard identification helps to identify chemical elements, which are present at specific locations in terms of their concentration and spatial distribution. The exposure assessment estimates the intensity, frequency, and duration of human exposure to environmental contaminants [27]. The risk assessment process would be used to estimate the average daily intake (ADI) of contaminants through ingestion, inhalation, and dermal contact by adults and children in the study area. The dose–response or toxicity assessment estimates the toxicity due to exposure levels of chemicals. The cancer slope factor (CSF, a carcinogen potency factor) and the reference dose (RfD, a non-carcinogenic threshold) are important toxicity indices used in risk estimation [9, 27]. RfD values are calculated from animal studies using the “No observable effect level” principle and for humans [24]. Risk characterisation helps to predict the potential cancerous and non-cancerous health risks for children and adults by integrating all the information gathered to arrive at quantitative estimates of cancer risk and hazard indices [9, 11, 28]. The potential exposure pathways for heavy metals in contaminated environmental media are calculated based on the recommendations by several American publications.

## Methods/design

A convergent parallel mixed-method design will be used to collect, analyse, compare, and merge qualitative and quantitative data to understand the research problem [29]. The convergent parallel mixed-method process is summarised in Table 1. This design is appropriate since qualitative and quantitative data would complement each other, help explain the health risks associated with potentially toxic elements, and the development of a health risk management framework. The study has two phases, and the first phase will involve gathering data from the environmental and community health surveys. The second phase will use the data gathered in phase one to inform the development and validation of the health risk management scheme.

**Table 1:** Outline of convergent parallel mixed-method design process

Phase	Method	Objectives	Participants	Data collection methods	Data analysis
Phase 1-Convergent parallel mixed method	Systematic literature review	1. Review literature on potentially toxic chemicals, health risks, and risk management frameworks	Studies and reports published in English from 2002 to 2022 in peer-reviewed journals	Data following the 4-step PRISMA guidelines	Thematic analysis
	Quantitative study	2. To determine levels of heavy metals and cyanide in surface water, soil, and vegetables	Soil, surface water, and vegetable samples	Grab samples to be analysed in a laboratory using FAAS	ANOVA, PCA, and correlation analyses in R Studio
		3. To characterise health risks associated with exposure to heavy metals and cyanide in environmental samples	US EPA risk assessment method	USEPA method to be used to characterise health risks	Statistical analysis in R
		4. To model data on heavy metals and cyanide levels, and health conditions using the Akaike information criterion (AIC) and the data’s relevance for the framework development	Models will be generated using AIC to determine the best fit for data	Data from environmental samples and health conditions to be collected from the community health survey	
	Qualitative study	5. To explore community health problems associated with heavy metals and CN contamination	Health practitioners, EMA officers, teachers, residents, and community leaders	Data collected using focus group (FGD), and interview guides	Thematic analysis in NVivo 12 software
		6. To assess the legal framework or schemes for the prevention and control of toxic metal/cyanide pollutants in Zimbabwe	Acts of parliament, regulations, and by-laws	Review of statutes on heavy metals and cyanide pollution	Thematic analysis

**Fig. 1:**
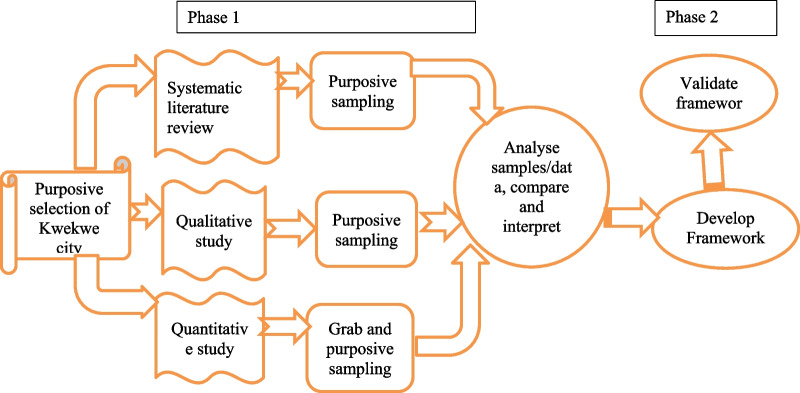
Research approach for developing a management framework for heavy metals and cyanide risks

Mixed-method research approach would be used in the study and will enable the researcher to collect, analyse, and integrate both quantitative and qualitative data in the study as shown in Fig. 1.

### Setting

**Fig. 2:**
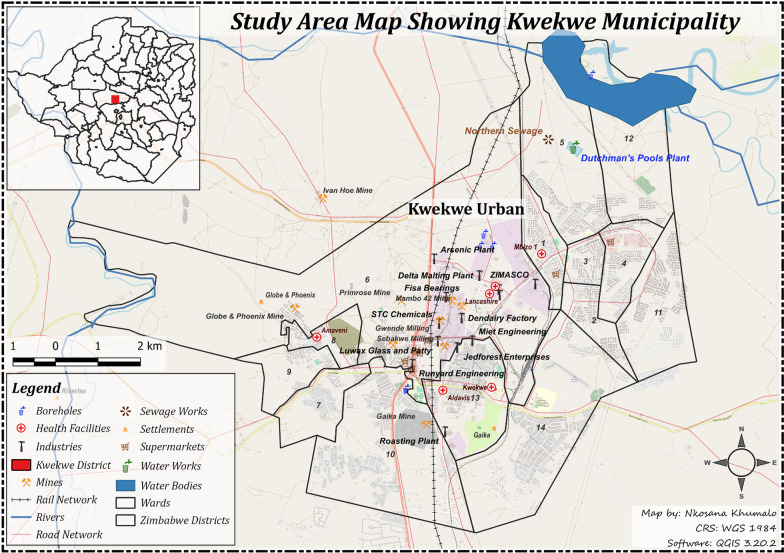
GIS Map of Kwekwe City, Zimbabwe (Nkosana Khumalo)

Kwekwe city lies between 18° 92′ S latitude and 29° 81′ E longitude and was established in 1898 by the British South African Company as a mining town due to the large gold deposits in the area. The geology of the area is known as the Rhodesdale gneiss terrain, which underlies the entire city [30]. Globe and Phoenix Gold Mine, which was started in 1894 by Edward Thornton and Joseph Schukala [31], spearheaded the industrial development of Kwekwe city to its present position as one of Zimbabwe's mining and industrial centres as shown in Fig. 2. Kwekwe city was purposively chosen as the study area due to its land-use activities, which include mining, metallurgical processing and heavy industrial manufacturing. Artisanal gold mining is widespread in the area, and many parts of the city bear scars of unregulated mining in the form of dumps, open pits, and damaged infrastructure. Large quantities of mercury and cyanide are used to extract gold from the ore, and much of these chemicals end up in the environment.

### Phase 1(a): systematic literature review

#### Review title: risk management frameworks for potentially toxic chemical pollutants: a critical review

##### Background of the review

Traditionally, statutes and legal practices in environmental pollution have tended to dictate management approaches that focus on risks posed by a single chemical in a specific medium [32–35]. Inferences about risks were based almost exclusively on observations of toxicity from high doses of chemicals in laboratory animals or the workplace. In reality, the exposure of humans to chemical mixtures is the rule rather than the exception, and therefore, health risk assessments should focus on chemical mixtures and not on single chemicals [25, 36]. It is true, however, that humans have learned to cope with exposure to various chemicals, simultaneously [25]. The toxicological data derived from exposure to different chemicals and environmental media, which is critical in the adverse outcome pathway (AOP) concept, provides a structured basis for clustering substances into risk assessment groups, identifying and collecting relevant toxicity data and identifying possible key upstream events used to calculate relative potency factors [25]. Many studies carried out in the last decade on potentially toxic metal and cyanide pollution made recommendations around legal and regulatory refinement or enforcement, but this has not adequately addressed the problem. While traditional approaches have tremendously contributed to reducing health, safety, and environmental risks in the past, they are inadequate for resolving more complex risks [4, 11, 35]. Therefore, creative and integrated approaches that address multiple environmental matrices and sources of risks are needed to sustain and strengthen environmental improvements, and for risk reduction. A health risk management framework is critical for risk decisions related to setting standards, controlling pollution, protecting health, and cleaning up the environment [4, 9, 11, 34, 35].

##### Review question

What are the global chemical risk management frameworks in use, their strengths, and shortcomings?

### Inclusion/exclusion criteria

Peer-reviewed articles and grey literature would be identified, screened, assessed for eligibility, and included in the review using the four-step Prisma flow chart [37, 38]. A critical review of literature from relevant publications, studies and reports written in English from 2002 to 2022 would be done. The scope of the review would focus on potentially toxic chemical elements, associated health risks, and management frameworks. The reviewed literature will exclude studies on the benefits of heavy metals or cyanide.

### Review methods

Titles and study abstracts would be reviewed first before extracting full articles and documents [38]. The researchers will review titles and abstracts to identify research articles and documents or reports that would be relevant to this systematic review. Key words in the search strategy include risk management framework; potentially toxic metals; cyanide; human health risks and risk assessment. These would be used to search for literature from databases such as EBSCO, Science Direct, PUBMED, Google Scholar, and Web of Science. To maintain high quality and academic standards, which form the foundation of literature review studies, only peer-reviewed articles published in highly reputable journals (indexed by Medline, Scopus, and Scimago) would be reviewed. Also, risk management framework documents (grey literature) developed by governments and private organisations would be reviewed. The AMSTAR tool will be utilised to assess the methodological quality of systematic reviews [39].

### Data extraction and synthesis

After the initial title and abstract screening, the selected articles and documents will be retrieved using a standardised protocol to extract the following information: management framework or approach, general description of the framework, potentially toxic chemicals, and references. The risk of bias will be reduced by focusing on the methodological criteria described above.

### Phase 1(a): quantitative study

An analytical cross-sectional survey would be used to determine levels of heavy metals in surface water, soil, and vegetables. Free cyanide will be determined in surface water samples only. Considering the huge and diverse number of toxic substances and their possible combinations, it is very important to identify and prioritise substances, and mixtures of concern to effectively manage them [27, 32]. Potentially toxic chemical pollutants associated with industrial activities in the area would be determined. Most of the industrial activities in Kwekwe revolve around base metal and gold mining, ferro-chrome smelting, and processing. This quantitative study would involve health risk assessment associated with identified heavy metals and cyanide. Environmental sampling will be done as per the British Columbia Sampling Manual within 8 to 12 months. Sampling sites would be selected based on their easy accessibility and representativeness [40]. The soil and vegetable samples would be prepared and analysed following the Method 3050b [41]. Water samples would be prepared and analysed following the EPA method 3005a. Reference samples for soil, surface water, and vegetables would also be collected outside the industrial and mining sites.

### Soil sampling, preparation, and analysis

Two hundred grams of triplicate samples would be collected at depths of 0–15 cm from stream beds, roadsides, footpaths, school grounds, parks, and rooftops using a garden trowel. The triplicate samples will be homogenously mixed, quartered, and placed in sealable polythene bags. The samples will be placed in a cooler box with dry ice during field trips en route to the laboratory, where they will be frozen at 4 °C until required for analysis [40]. Soil sample preparation would involve defrosting, air-drying at 30 °C, and thorough mixing. 0.1 g of each sample will be measured and mixed with 3 mL of nitric acid (HNO_3_) in a 10-mL borosilicate vial. The mixture is left at room temperature to allow a pre-digestion of the contents. The glass vial is then capped and placed in hot block at 120 °C. After 1 h, 1 mL of 30% hydrogen peroxide (H_2_O_2_) is carefully added and heated for one more hour at 120 °C. The digested sample is then diluted 3 times, and each sample is then measured 2 times and 2 blanks are processed as well. The samples would be analysed using flame atomic absorption spectrometry (FAAS) following EPA method 3050B. The rationale for using this method is that it has specificity for the accurate detection and quantification of heavy metals [41].

### Surface water sampling, preparation, and analysis for total dissolved metals

Samples would be collected from streams and other water bodies in the industrial areas. One-litre sampling bottles would be washed thoroughly, soaked in 10% HCl for 24 h, rinsed with deionised water, and dried [40]. Before sampling, the bottles will be rinsed quantitatively with water to be sampled. Triplicate surface water samples will be randomly collected at water depths of 30–80 cm within a distance of 20–50 m from each sampling site. The individual samples from a site will be equally and homogenously mixed to form one and then split into two samples, measuring one litre each. At the sampling site, the split water samples would be filtered through a 0.45-µm filter to remove turbid and suspended matter. The one sample for metal analysis would be preserved by adding 0.3 mL 65% HNO_3_ to attain a pH below 2 to prevent adsorption and crystallisation of metals before analysis and then stored at 4 °C [41]. During the field trip, a field bank made of distilled water will be treated in the same manner as the experimental water samples [40]. The samples would be analysed for total dissolved heavy metals using FAAS [41].

### Surface water sampling, preparation, and analysis for free cyanide test

50% (10 M) sodium hydroxide is added to the other split water sample (pH is adjusted to 12) is stored in the dark at 4 °C in a cooler box. The sample will be stored under these conditions for up to 14 days before analysis. Cyanide determination would be determined using the ion-selective electrode [41]. 0.5 ml of sodium hydroxide (ionic strength adjustment solution, ISA) would be added to 50 ml of the water sample in a 100-ml beaker and a PTFE-coated magnetic stirrer would be added to the sample. The beaker would be placed on a magnetic stir plate and stirred at a slow speed. The tip of the electrode is immersed just above the rotating stirrer. The meter is read (in millivolts or concentration) as soon as the reading stabilises within 5 min of immersing the electrode tip. If the reading is in millivolts, CN concentration is determined from the calibration curve.

### Vegetable sampling, preparation, and analysis

Four types of vegetables (leafy, tubers, fruit type, and legume-like) commonly grown and consumed in the study area would be hand-picked in triplicates. The samples would be washed with tap water and rinsed using distilled water to remove any attached soil particles, then placed in clean sealable polythene bags and kept on dry ice in the field. The samples would be stored frozen in the laboratory until required for analysis. When ready for analysis, the samples would be allowed to defrost and then ground using a pestle and mortar into a fine powder. The triplicate vegetable samples would be weighed (0.1 g) into glass vials and 3 ml concentrated HNO_3_ will be added. The preparation and analyses would proceed as in the EPA method 3050b used for the soil samples. The results would be reported in mg/kg dry weight of the sample.

### US EPA health risk assessment procedure

To estimate the potential health risks associated with long-term ingestion of food contaminated with toxic elements, the average daily dose (ADD) of contaminant, hazard index (HI), target hazard quotient (THQ), and non-carcinogenic risk (NCR), and carcinogenic risk (CR) will be calculated [9]. ${\text{ADD}} = \frac{{\left( {{\text{Ci}} \times {\text{IR}} \times {\text{EF}} \times {\text{ED}}} \right)}}{{{\text{BW}} \times {\text{AT}}}}$, where Ci is contaminant concentration, IR is ingestion rate, EF is exposure frequency, ED is exposure duration, BW is consumer body weight, and AT is the average time. The health risk is assessed with its non-carcinogenic and carcinogenic effects based on the calculation of ADD estimates and defined toxicity [9, 12, 24]. The estimation of the non-carcinogenic risk of contaminant consumption is determined using THQ values. The target hazard quotient is a ratio of the determined dose of a contaminant to the oral reference dose considered detrimental. An exposed population is at risk if the ratio is greater than or equal to 1. The non-carcinogenic risk is calculated as follows: ${\text{HQ}} = \frac{{{\text{ADD}}}}{{{\text{RfD}}}}$ where RfD is the reference dose. The HI estimates the potential human health risk when more than one toxicant is involved and is calculated as the sum of THQs. Carcinogenic risk assessment is the incremental probability of an individual developing cancer over a lifetime due to exposure to a potential carcinogen [12, 23, 24, 28]. The equation for calculating the lifetime carcinogenic risk is: ${\text{Riskpathway}} = \mathop \sum \limits_{k = 1}^{n} \left( {{\text{ADDk}} \times {\text{CSFk}}} \right)$.

Risk is a probability (without units) of a person developing cancer over a lifetime [12]. ADDk (mg/kg/day) and CSFk (mg/kg/day) are average daily doses and the cancer slope factor, kth toxic element, for number (n) of elements. The slope factor converts the estimated daily dose of the toxic element averaged over a lifetime of exposure directly to the incremental risk of an individual developing cancer [9]. The total excess lifetime cancer risk for an individual is finally calculated from the average contribution of the individual elements for all the pathways using the following equation: ${\text{Risk}}\left( {{\text{total}}} \right) = {\text{Risk}}\left( {{\text{inh}}} \right) + {\text{Risk}}\left( {{\text{ing}}} \right) + {\text{Risk}}\left( {{\text{dermal}}} \right)$, where Risk (inh), Risk (ing), and Risk (dermal) are risk contributions through ingestion, inhalation, and dermal pathways, respectively [12, 23].

### Reliability and validity

Certified analytical-grade reagents would be procured from reputable suppliers. For quantification and detection limits of the FAAS, a blank solution will be read several times, and standard deviations will be considered for the noise generation level for each analyte [41]. To check for the reproducibility and accuracy of the analysis, each sample will be measured in triplicate. Certified reference materials (CRMs) and standard reference solutions with known concentrations of elements [23, 41] will be used to assure quality and establish the accuracy of the measurements using the FAAS and ion-selective electrode method.

### Data collection and analysis data

The data on environmental sample analysis obtained from the FAAS would be printed on tabulated spreadsheets from an interfaced computer and stored until required for analysis. The analysis of quantitative data would be done using descriptive and inferential statistical methods in R [12, 23]. The one-way ANOVA, principal component analysis (PCA), Pearson correlation, and Akaike information criteria (AIC) will also be performed on data. One–way ANOVA would be used for statistical analysis since there would be one independent variable and one dependent variable. The PCA would be used to establish the main variables associated with specific environmental compartments and the contribution of variables to data variance. The Pearson correlation analysis would measure the linear correlation between environmental pollutants. The AIC would elaborate on the variables which are important for predicting the relationship between levels of environmental pollutant, and the reported community health problems [38].

### Phase 1(c): qualitative study

The phenomenological qualitative inquiry will be used to investigate events or phenomena around health risks associated with environmental pollutants to describe or interpret participants' lived experiences [29]. The study aims to understand the participants' health problems, health status, behaviours, and attitudes regarding health risks associated with heavy metals and cyanide pollution [29, 42]. The qualitative study will augment the quantitative study by gathering complementary data from the study population, which is critical in data triangulation. This research design would also help to identify local health problems and potential solutions that could facilitate the development of a framework to prevent and control specific health problems to improve health outcomes. The qualitative research process is outlined as shown in Table 2

**Table 2:** Research process outline for the qualitative study design

Participants	Sampling method	Sample size	Measurement instruments	Data collection procedures	Data analysis
Health service providers	Purposive	The study area has 22 health facilities and at least 2 health workers (doctors, nurses, and environmental health practitioners) from each facility would be selected, however, the sample size would depend on data saturation	Unstructured interview guide	Note-taking, tape recording, and photographs	Data transcription, coding, and thematic analysis using N-Vivo 12 software
Community leaders	Purposive	At least 5 councillors from study areas, 10 headmasters/teachers, 7 council managers, and 2 EMA officials would be recruited, however, the sample size would depend on data saturation	Unstructured interview guide	Note-taking, tape recording, and photographs	Data transcription, coding, and thematic analysis using N-Vivo 12 software
Residents	Purposive	Five focus groups of 5 to 8 participants comprising an equal number of males and females aged between 18 and 65 years would be recruited	Focus group discussion (FGD) interview guide	Note-taking, tape recording, and photographs	Data transcription, coding, and thematic analysis using N-Vivo 12 software

The city has 22 health facilities and at least 2 health workers (doctors, nurses, and environmental health practitioners) from each facility would be selected, however, the sample size would depend on data saturation.

### Study population

The health service providers, community leaders, environmental regulatory officers, and local authority managers would be recruited as key informants. The residents living in or near mining and industrial sites would also be recruited for the study. The participants of mixed gender and in equal proportion will be purposively selected. The health providers would include nurses, environmental health practitioners, and medical doctors working or resident in study areas. Community leaders would include councillors, and headmasters or teachers.

### Sampling

Kwekwe city was purposively chosen as the study site due to mining and heavy industrial activities, which are prevalent in the area. Purposive sampling will be used to select key informants and residents to participate in the study. The sample size for key informants to be interviewed would be determined by data saturation, while 5–8 residents from 5 study sites would be selected for focus group discussions. The sampling process for the target population is summarised in Table 2.

### Sample size and sampling

The participants would be selected using the purposive sampling method [38]. Key informants in study areas will be visited at their workplaces during normal working hours, and permission to interview them will be sought from heads of their institutions or gatekeepers. The health workers from each of the twenty-two health facilities in the study area would be purposively selected. Health workers are often involved in information dissemination and public health programmes, thus more knowledgeable about health events in their communities [29]. The teachers and lecturers drawn from 7 schools and colleges in the study area will also be recruited at their workplaces and homes. The local authority councillors and officials, and EMA officers are familiar with local environmental and health problems in their areas; hence, they would also be recruited. The residents would be recruited from their homes and or workplaces in wards 2, 5, 6, 7, 10, and 13, which are areas where mining and industrial activities are prevalent. However, the sample size for each group will be determined by data saturation. Five focus groups of 5–8 participants from mining and industrial areas, comprising an equal number of males and females would be chosen. All ethical protocols will be adhered to in the selection of research participants.

### Inclusion/exclusion criteria

Health providers, EMA and council officials, teachers and residents from different parts of the study area would be eligible to participate in the study. The justification for their inclusion in the study is premised on their knowledge of environmental health issues and lived experiences. Participants aged between 18 and 65 who would have stayed in the study area for at least 6 months would participate in the study since they are perceived to have a better understanding and recall of past environmental or health events. Residents who are bed ridden and the mentally sick will be excluded from the study since they may have recall problems or may not fully comprehend the aspects of the study, especially health risks associated with heavy metals and cyanide.

### Measurement instrument

The focus group discussion guides, unstructured interview guides and checklists would be used by the researcher for data collection over a period of 6–8 months. The unstructured interview and focus group discussion guides will be used to obtain data from key informants and residents. Each focus group discussion will be conducted for an average period of 45 min, and interviews will be conducted for an average of 15 min per person. The residents would participate in FGDs, and each of the 5 groups would comprise 5–8 participants. The unstructured interview guides and checklists would be in languages commonly used in the area, which include English, Shona, and Ndebele. Probing questions will be asked to seek clarity on issues that would arise during interviews and focus group discussions [38]. A pretest of the tools will be conducted in Globe and Phoenix Mine (in Ward 6) to avoid contamination of sampling pools. Three residents from the community, three from health facilities, and two from EMA would be selected to participate in unstructured interviews and one focus group discussion. The responses from the pretest would be transcribed and analysed. Appropriate adjustments would be made to the FGD and interview guides, but the results of the pretest would not be reported.

### Data collection and analysis

Unstructured interviews would be administered on key informants to understand public and environmental health problems associated with heavy metals, and cyanide. The interviews and focus group discussions would focus on the nature of health problems associated with toxic metal and cyanide pollution that is encountered by the community, nutritional issues, modes of exposure to heavy metals and their sources, and possible solutions to the problems. Note-taking, tape recording, and photographs would be taken during FGDs and interviews after obtaining permission from participants. For data analysis, the following steps would be taken: familiarising with the data, generating initial codes, searching for themes, reviewing themes, defining themes, and writing up [29]. Themes are patterns across data sets that are important in describing a phenomenon and are associated with a specific research question. The data (that would have been obtained in Shona or Ndebele) collected from respondents would be transcribed, translated to English, and clustered into main themes, categories, and sub-categories using N-Vivo 12 statistical software. The themes would be presented as findings that would be interfaced with results from the quantitative study.

### Trustworthiness of the study

Trustworthiness is a function of credibility, transferability, dependability, and conformability and is critical in ensuring the data's robustness [29, 38]. The use of different data collection instruments and various categories of respondents through FGDs and interviews would contribute to research credibility [38]. Also, prolonged engagement, peer debriefing, negative analysis, and member checking are key to ensuring research credibility [29]. Transferability refers to the extent to which a study's findings can be generalised to a broad population or applied to different settings. Expert involvement and method/data triangulation are critical in ensuring the transferability of the study. Dependability refers to a process of evaluating the quality of integrated processes of data collection, analysis, and theory generation [29]. The use of a parallel mixed-method convergent study to collect and analyse data would strengthen the reliability of the study. The data collection and analysis strategies will be comprehensively provided to ensure a clear and accurate picture of the methods used in this study. Also, ethical clearance would be sought from relevant bodies and the research findings would be presented to experts and key stakeholders as a way of validation. In order to ensure conformability of findings, manuscripts would be submitted to peer-reviewed journals to enable reviewers to scrutinise the methods and data analysis techniques used to collect, analyse and interpret data [29, 38].

### Merging of quantitative and qualitative data

The researcher would merge quantitative and qualitative data by separately comparing, analysing, and interpreting the findings [29]. This would show that the two data sets bring about convergence or divergence [29, 38]. If there is divergence, the researcher would follow up or indicate that as a limitation. Alternatively, the researcher may revisit the data as this might be an indication of errors in data collection.

### Phase 2(a): development of a health risk management framework

This phase will involve the development of a framework that would assist in managing health risks associated with potentially toxic chemicals, with a focus heavy metals and cyanide. The results from both qualitative and quantitative studies would be used to develop the framework for managing heavy metals and cyanide health risks in the community. As outlined in Table 3, the development of the health risk management framework would involve applying a SWOT analysis, a logical framework, and a multi-level intervention model [43–45].

**Table 3:** Research process** o**utline for the development and validation of risk management framework

Phase	Method	Objectives	Participants	Data collection methods/techniques	Data analysis
Phase 2-Development and validation of the developed framework	Development of a management framework	1. To develop a framework using empirical evidence to manage health risks associated with HMs and CN	1. Data analysis from environmental sample analysis and participants in phase 1	SWOT matrix, Log frame and multi-intervention model	SWOT analysis would be used to identify internal and external factors, threats and opportunities that are key to the implementation of the management framework
	Validation of the developed framework	2. To validate the developed Framework	Experts and key stakeholders	Checklists	Qualitative analysis of experts’ and stakeholders’ feedback data

The Strengths, Weaknesses, Opportunities, and Threats (SWOT) analysis is an organised process that aims to identify and analyse the strengths, weaknesses, opportunities, and threats to the framework designed to achieve specific objectives [38]. A deeper understanding of health risks posed by heavy metals and cyanide would be obtained from the merged qualitative and quantitative data. The SWOT technique would be applied to the findings to develop practices, systems, and policies that would be critical in managing pollution-related anthropogenic and environmental factors. The SWOT analysis would assist in identifying and analysing internal and external factors that are key in developing the management framework. This would form the basis of framework concept brainstorming and appraisal using the logical framework (log frame). Findings obtained from the SWOT analysis would then be scrutinised using the log frame, which will be used to guide the development of the health risk management framework based on SWOT analysis findings. The log frame is a set of interlocking concepts utilised dynamically to develop a well-designed, objectively described, and valuable project [43]. The log frame defines four basic levels of responsibility, which include inputs (resources and activities), outputs (products or results), purpose (reason for producing outputs), and goal (higher-level objective).

The multi-level intervention model would complement the log frame in developing a health risk management framework. The multi-level intervention approach is the application of integrated and coordinated programs that intervene in multiple community settings or institutions and involves policy and systems changes [46]. The multi-level interventions work on more than one level at the same time and involve more intervention components that are coordinated across levels [46]. The developed framework would enable the prevention, reduction, and control of health risks at different levels. The empirical findings from phase 1 would provide a basis for understanding the health risks at the individual, interpersonal, organisational, community, and societal levels or a combination of these levels.

### Phase 2(b): validation of health risk management framework

Validation is intended to determine the feasibility, applicability, acceptability, and sustainability of the developed framework in achieving desired goals [38, 47]. As outlined in Table 3, the validation would involve the evaluation of the developed framework by key stakeholders to ensure that they do not undermine or violate the values of different risk management system users. The framework evaluation would be done in two stages. The first stage would be done using the Delphi technique, and the second stage would involve the administration of checklists on key stakeholders. The validation of the framework would include applying Delphi Technique and using an adapted checklist that would be administered to experts and key stakeholders to get their opinion on the developed framework [47].

### The Delphi technique

This would involve experts' forecasting and assessing if the proposed risk management framework would meet future objectives [47, 48]. The method relies on experts who are knowledgeable about the development of frameworks and would be asked to forecast the outcome of future scenarios, and predict the effectiveness of the developed framework. The aim is to develop an expert-based judgement about the proposed framework [47]. Based on framework objectives, the Delphi technique would aim to reach a consensus, aggregate ideas, make future predictions, and determine experts' opinions. The selection criteria for experts in the Delphi process would be based on organisational or institutional affiliation, recommendation by third parties, and experience [47, 48]. Fifteen experts in the field of health systems, environmental health, policies, monitoring and evaluation, and management framework development in the country would be purposefully recruited. The experts would be briefed on the findings from the convergent parallel mixed method study, SWOT analysis, log frame, multi-intervention model, and the developed framework. The team of experts would critique and evaluate the developed framework using a checklist and offer feedback after responding to checklist questions.

### Feedback to stakeholders

It is critical to involve key stakeholders in crafting risk management frameworks from the onset to ensure that they accept and implement risk management decisions they would have been involved in shaping [11, 42]. Hence, findings would be consolidated, thematically presented, used to refine the framework, and presented to key stakeholders for input [38]. A checklist would be used to gather data on key stakeholders' opinions on the feasibility, accessibility, and sustainability of the proposed framework. Their feedback would be analysed and used to fine-tune the management framework.

## Discussion

Limited peer-reviewed studies on risk management frameworks were found in the literature. Most of the identified quantitative studies focused on the determination of levels of heavy metal contaminants, pollutants' spatial distribution, pollution indices, statistical analysis, and health risk assessments. The empirical studies found in the literature did not develop frameworks to manage toxic elements like metals or cyanide. While existing risk management frameworks have contributed significantly to human and environmental health protection, novel and comprehensive frameworks need to be developed to counter the ever-dynamic and evolving risks associated with chemical substances and pollutants [4, 11]. This study might help to identify health risks, explore community health problems, and develop a management framework for heavy metals and cyanide pollution in Kwekwe city.

## Conclusion

The complementarity of quantitative and qualitative inquiries in data triangulation, which might help to identify toxic metal and cyanide pollutants, and diagnose community health problems are critical in informing the development of a risk management framework. If the management framework is successfully developed, it could offer an opportunity to prevent and control heavy metals and cyanide pollution in the city. This study is expected to generate at least four publications in peer-reviewed journals.

## Data Availability

Not applicable.

## Abbreviations

As: Arsenic
ASGM: Artisanal small-scale gold mining/miners
Cd: Cadmium
Co: Cobalt
CN: Cyanide
Cr^6+^: Chromium VI
Cu: Copper
DNA: Deoxyribonucleic acid
EMA: Environmental Management Agency
FAAS: Flame atomic absorption spectrometry
Fe: Iron
FGD: Focus group discussion
H_2_SO_4_: Sulphuric acid
HCl: Hydrochloric acid
Hg: Mercury
HIV/AIDS: Human immunovirus/acquired immunodeficiency syndrome
HM(s): Heavy metal(s)
HNO_3_: Nitric acid
IARC: Agency for Research on Cancer
NHMRC: National Health and Medical Research Council
Pb: Lead
USEPA: United States Environment Protection Agency
WHO: World Health Organization
Zimasco: Zimbabwe Iron Smelting Company
ZIMSTAT: Zimbabwe National Statistics Agency
ZINWA: Zimbabwe National Water Authority
Zn: Zinc
